# Regionalization of Daily Soil Moisture Dynamics Using Wavelet-Based Multiscale Entropy and Principal Component Analysis

**DOI:** 10.3390/e21060548

**Published:** 2019-05-30

**Authors:** Yuqing Sun, Jun Niu

**Affiliations:** Center for Agricultural Water Research in China, China Agricultural University, Beijing 100083, China

**Keywords:** soil moisture, variability, wavelet transform, multiscale entropy, Xijiang River

## Abstract

Hydrological regionalization is a useful step in hydrological modeling and prediction. The regionalization is not always straightforward, however, due to the lack of long-term hydrological data and the complex multi-scale variability features embedded in the data. This study examines the multiscale soil moisture variability for the simulated data on a grid cell base obtained from a large-scale hydrological model, and clusters the grid-cell based soil moisture data using wavelet-based multiscale entropy and principal component analysis, over the Xijiang River basin in South China, for the period of 2002–2010. The effective regionalization, for 169 grid cells with the special resolution of 0.5° × 0.5°, produced homogeneous groups based on the pattern of wavelet-based entropy information. Four distinct modes explain 80.14% of the total embedded variability of the transformed wavelet power across different timescales. Moreover, the possible implications of the regionalization results for local hydrological applications, such as parameter estimation for an ungagged catchment and designing a uniform prediction strategy for a sub-area in a large-scale basin, are discussed.

## 1. Introduction

**Figure 1:**
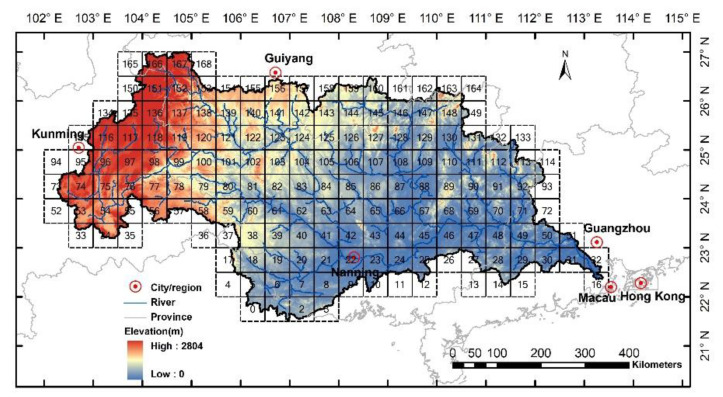
The Xijiang River basin in South China and the examined 169 grid cells in this study.

The Xijiang River basin (Figure 1) is the largest tributary of the Pearl River basin in South China, with a total basin area of 0.35 million km^2^ [1,2,3]. Recently, with high-speed social and economic development in the Pearl River Delta, large water demands are required from the new modern cities, such as Shenzhen and Zhuhai. However, the water amount from the Dongjiang River and Beijiang River basins (the other two major tributaries in the Pearl River) may be not able to meet the various kinds of water demands in the region, especially in the dry years. One large water diversion project, which has already been proposed and approved, aims to divert water from the downstream Xijiang River to the big cities. Therefore, the variability in the amount of water over the Xijiang River basin is extremely important for the regional water transfer, water allocation, and other water-related management issues. Accordingly, a series of hydrological simulations and predictions under the changing environment are prerequisite to the water resource management in the basin. 

For a large-scale basin, the homogeneity issue is a common problem when we perform the modelling of terrestrial hydrological processes and the relevant dynamic predictions. The catchment regionalization is an important step [4], such as when estimating hydrological parameters of the ungagged basin [5], selecting optimal models for local hydrological variable predictions, and studying the possible runoff response features [6]. Meanwhile, the regionalization of hydrological variables should be more appropriate across different scales, as its evolution incorporates the results of many surrounding factors [5,7,8]. Among them, the divergences of water resource variables related to time-space scales could be quantified by the approaches presented in [9].

There are many different approaches in hydrological data analysis, such as multivariate analysis for the factor identification [10], a genetic algorithm for the global optimization technique [11], a neural network-based model for the probability assessment [12], the knowledge-based clustered partition technique [13], among others. As the hydrological variable is a product of integrated effects of many forcing and affecting factors, which certainly occur at a range of time and space scales. Meanwhile, the effective summarization of intermittency and time variability at a given scale is also important. Therefore, the multi-scale analysis approach would be more appropriate.

Regarding the multi-scale analysis, the wavelet transform is a powerful tool to decompose a time series from a signal time domain to a time-frequency domain. The advantages of a wavelet transform have been demonstrated in many hydrological regionalization studies e.g., [7,14] (Shannon proposed the entropy theory in the 1940s [15] and Jaynes presented the principle of maximum entropy in the 1950s [16,17], which have been employed in many different research fields). The applications of entropy theory in hydrology science are also noticeable e.g., [18,19,20,21,22,23,24] (Agarwal et al. [5] proposed the wavelet-based multi-scale entropy method to give an entropy signature across multiple scales for a streamflow time series over the contiguous United States. The wavelet-based multiscale entropy can capture the information of the embedded evolution processes, as it is an effective measure of the disorder degree of the signal across different timescales, which is useful in regionalization studies [5]. The present study examines the multi-scale variability of top 1-m grid-based soil moisture data [25] for the Xijiang River basin in South China, performs the classification using wavelet-based multi-scale entropy, and discusses the possible applications of the obtained soil moisture regionalization results.

This paper is organized as follows. The methods and analyzed data are introduced in Section 2 and Section 3, respectively. The multi-scale soil moisture variability, the grid-based hydrological regionalization with the wavelet-based entropy information, and the possible applications of the obtained results are presented in Section 4. The conclusions are given in Section 5.

## 2. Data and Methodology

**Figure 2:**
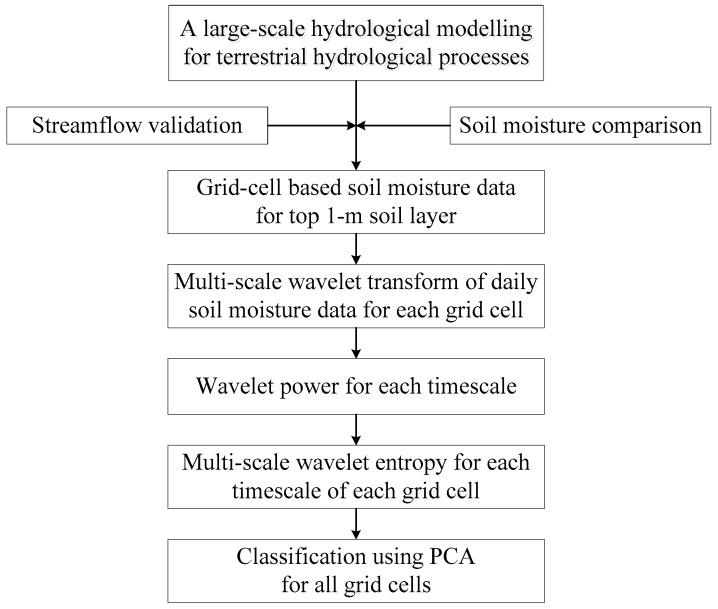
The schematic methodology flowchart in this study.

The analyzed time series (i.e., daily soil moisture data with the spatial resolution 0.5° × 0.5°) are derived from the large-scale hydrologic modelling using the Variable Infiltration Capacity (VIC) model [26,27] for the terrestrial hydrological processes in the Xijiang river basin in South China. The grid-cell based simulations have been validated by both the records of streamflow gauging stations and the reanalysis of soil moisture data. The wavelet transform is employed to reveal the multi-scale variability embedded in the daily top 1-m soil moisture time series, and the wavelet-based entropy is used to unfold the disorder features of each examined timescales. The principal component analysis is subsequently performed on the multi-scale wavelet entropy to regionalize all grid cells over the studied basin. The whole methodology is illustrated in Figure 2. 

### 2.1. Data

The soil moisture data for studying temporal and spatial variability and the hydrological regionalization in the present study are obtained from VIC modelling over the Xijiang River basin in South China, for the period of 2001–2010. The simulations of terrestrial hydrological processes over the basin have been reported in the study reported by Niu et al. Among them, the daily forcing data, including precipitation, maximum/minimum temperature, and wind speed, for the period of 2001–2010, were obtained from 32 national standard weather stations over the studied area and further gridded to 0.5° × 0.5° grid cells. The year 2001 served as the model spin-up period, then the simulated daily top-1 m soil moisture data for the period 2002–2010 are derived for 169 grid cells for the present study. For the modelling of the terrestrial hydrological processes in the Xijiang River basin, the runoff simulations have been effectively validated by six gauging stations in the basin [3,28], and the comparisons between the simulated soil moisture and re-analysis data from the National Center for Environmental Prediction are also examined [28,29].

### 2.2. Wavelet Transform 

The wavelet transform has been applied in the field of hydrology in many cases (see [30]), as it is a powerful tool to analyze variability properties for both stationary and nonstationary time series at different timescales. Mathematically, a wavelet transform decomposes a time series *x_t_* in terms of “daughter” wavelets *ψ*(*t*, *s*) derived from a “mother” wavelet function *ψ*_0_(*t*) by the timescale (*s*) dilation and time position (*t*) translation:

(1) $$\psi (t,s)=\frac{1}{s^{1/2}}\psi_{0}(\frac{t^{\prime }-t}{s})$$

where s^1/2^ is an energy normalization factor to keep the energy of daughter wavelets the same as the energy of the mother wavelet. The wavelet transform of the time series *x_t_* is defined as the convolution integral of *x_t_* and a dilated and translated version of *ψ*_0_(*t*):

(2) $$W(t,s)=\frac{1}{s^{1/2}}\int \psi^{*}(\frac{t^{\prime }-t}{s})x_{t}dt$$

where ψ* is the complex conjugate of *ψ* defined on the time and scale.

In this study, considering a time series *x_t_* (i.e., simulated soil moisture) observed at an equal time interval *δt* (i.e., daily) over a period of time t = 1, …, T, for the purpose of convenience, the timescales of wavelet transform are written as fractional powers of two:

(3) $$s_{j}=s_{0}2^{j\delta_{j}},j=0,1,\ldots ,J.$$

(4) $$J=\delta_{j}^{-1}\log_{2}\left(\frac{T\delta_{t}}{s_{0}}\right)$$

where *s*_0_ is the smallest resolvable scale and *J* determines the largest scale [31]. The *δ_j_* is a parameter based on the width in spectral-space of the wavelet function. The smaller value of *δ_j_* will provide finer resolution. For the Morlet wavelet used, it is suggested that a *δ_j_* of about 0.5 is sufficient [31], and the 0.12 is adopted giving a total of 39 scales in this study.

### 2.3. Multi-Scale Wavelet Entropy

Multi-scale wavelet entropy is an effective tool to gage the complexity of a time series (such as the daily soil moisture time series in this study), with utilizing the wavelet transform and Shannon entropy methods collectively [5]. The entropy-based investigation on wavelet power at different timescales provides comprehensive information to determine the least-biased probability distribution of a random variable. A discrete form of entropy *H_wt_(x)* is written as (Shannon, 1948):

(5) $$H_{wt}\left(x\right)=-\overset{K}{\underset{k=1}{\sum }}p(x_{k})\;\log_{2}\left[p\left(x_{k}\right)\right]$$

where *k* is the time interval of the *K* events, *x_k_* is a wavelet variation corresponding to the interval *k*, and *p*(*x_k_*) is the probability of *x_k_*. *H_wt_(x)* is a measure of information content in the analyzed signal, a lower entropy reflects more information, and less information is represented by a higher entropy.

(6) $$P\left(x_{k}\right)=\frac{E\left(k,j\right)}{TE\left(j\right)}=\frac{{\left|W\left(k,j\right)\right|}^{2}}{\sum {\left|W\left(k,j\right)\right|}^{2}}$$

where *E*(*k*, *j*) denotes the wavelet energy at time position *k* and time scale *j*, *TE*(*j*) is the total wavelet energy of the soil moisture time series at timescale *j*.

A high value of *H_wt_* at certain time scales represents a high degree of highly complicated and disordered hydrological systems, which indicates a high degree of unpredictability embedded. More details about the entropy and its applications are provided in Singh [18].

The wavelet energy based entropy measure was proposed in [32,33], Agarwal et al. [5] proposed the wavelet-based multi-scale entropy for the hydrological regionalization. Wavelet-based multi-scale entropy is a useful measure of the order/disorder degree of the analyzed signal and reflects information associated with the multi-scale signal. The application in this study is to measure the spatial and temporal variability/disorder features of soil moisture in Xijiang River basin in South China.

### 2.4. Principal Component Analysis 

The principal component analysis transforms a high-dimensional dataset into a low-dimensional orthogonal feature space, but nevertheless represents a large fraction of the variability contained in the original dataset [34]. The application effectiveness on multi-scale hydrological variables have been demonstrated in the study of Niu et al. [6]. The principal component analysis in this study was employed to classify the coherent modes of the wavelet-based multi-scale entropy of the top-1 m soil moisture for a large number of grid cells. It transforms a high-dimensional (of N dimensions) dataset into a low-dimensional orthogonal feature (eigenvector) space (of M dimensions, with N > M), but nevertheless represents a large fraction of the variability contained in the original dataset [34]. In the present study, the high-dimensional dataset is the wavelet-based entropy at K (K = 39) scale for N (N = 169) grid cells with the spatial resolution 0.5° × 0.5°. The elements of the new M vectors (V = V1, …, VM) are referred to as the principal components (PCs).

The eigenvalues (λ) are scalar descriptions of the degree of variance explained by the corresponding PCs. In this study, the M (number of) PCs are determined using the following four steps: (1) The wavelet-based entropy of each grid cell is standardized by subtracting its mean and dividing by its standard deviation; (2) The covariance matrix *C_i,j_* (*i* = 1, 2, …, N; *j* = 1, 2, …, N) of the N wavelet-based entropy is computed; (3) A matrix of eigenvectors is obtained by decomposing the covariance matrix. The transformation conserves the total variance as:

(7) $$\sum_{i=1}^{N}C_{i,i}=\sum_{i=1}^{N}\lambda_{i}.$$

Both the Scree graph (eigenvalue versus PC number) and Kaiser’s rule [35] are used to determine how many PCs are retained (i.e., the value of M). 

## 3. Results

### 3.1. Multi-Scale Variability Of The Grid-Based Soil Moisture

**Figure 3:**
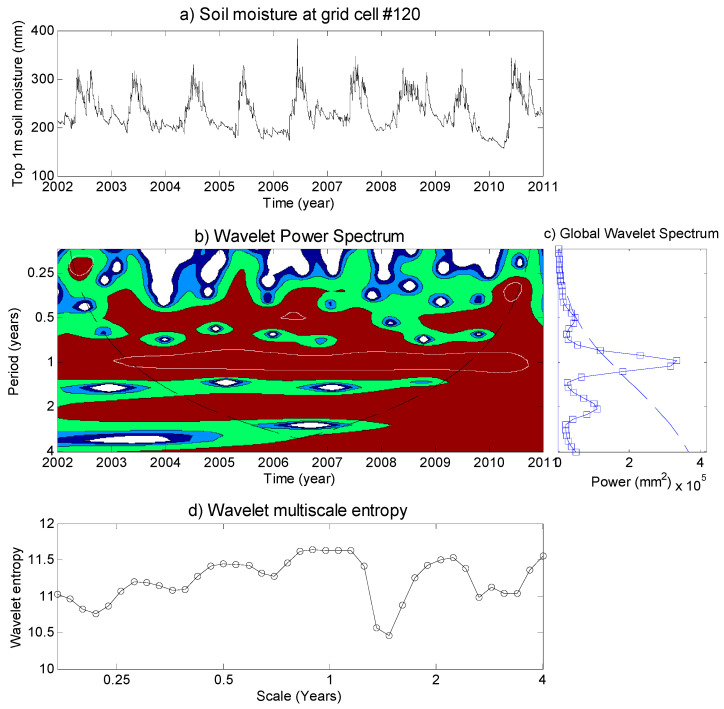
(**a**) The daily soil moisture time series for the 120^th^ grid cell in the Xijiang River used for the wavelet analysis. (**b**) Its local wavelet power spectrum, normalized by 1/σ^2^ (σ^2^ = 1324 mm^2^). The dashed curve depicts the cone of influence beyond which the edge effects become important. The color contours are at normalized variances of 1 (royal blue), 2 (blue), 4 (green), and 16 (red). The white contour closes regions of greater than 95% confidence for a red-noise process with a lag-1 coefficient α of 0.98. (**c**) Global wavelet power spectrum (solid line with square) over 39 timescales, and the dashed line is the 95% confidence level. (**d**) The corresponding wavelet multi-scale entropy over 39 timescales.

The multi-scale variability of the daily top 1-m soil moisture time series for the grid cell No. 120, using the continuous wavelet transform, is displayed in Figure 3. The local wavelet power spectrum in Figure 3b, ${\left|W\left(t,s\right)\right|}^{2}$, is normalized by 1/STD^2^ (with STD = 1324 mm). The vertical axis of Figure 3b is the wavelet timescale, and the horizontal axis is the time position for the period of 2002–2010. The values of $s_{0}=2\delta_{t}(60)$, $\delta_{j}$ = 0.11, and *J* = 38 in equation (3) and (4) give a total of 39 timescales ranging from 62 days to 4.01 years. The shaded contours are at normalized variances of different levels. The soil moisture variability at both the time and frequency domain are revealed by the local wavelet power. The significant regions highlighted by the white contour show less randomness of the dynamic soil moisture evolutions. As shown in Figure 3b, the high variability events of soil moisture are detected during the period of 2002–2003 around the 0.25-year timescale, the period of 2006–2007 around the 0.5-year timescale, and around 2010 in the band of 0.25–0.5-year timescales. The annual regular soil moisture variability is also identified by the local wavelet spectrum. 

Figure 3c provides the global wavelet power spectrum of the analyzed soil moisture time series and its 95% confidence level spectrum by assuming the red-noise processes with lag-1 autocorrelation. It is observed that the significant timescales are at some very short timescales, 0.5-year, and around 1-year. These are partly related to external effects of remote climatic patterns, as it is found be related to the evolutions of teleconnection patterns (e.g., Indian Ocean Dipole (IOD) and El Niño-Southern Oscillation (ENSO)), demonstrated in the study of Niu [36].

The wavelet power entropy results for the 39 timescales are shown in Figure 3d. The high value of entropy indicates the high disordered soil moisture at certain timescales, and a low value of entropy represents relatively consistent features at these timescales. As shown in the figure, those variabilities that are not very high in the global wavelet power spectrum may also show high disorder features, such as in the band of the 0.25–0.5-year timescale, and the annual soil moisture shows both high variability and disorder characteristics.

**Figure 4:**
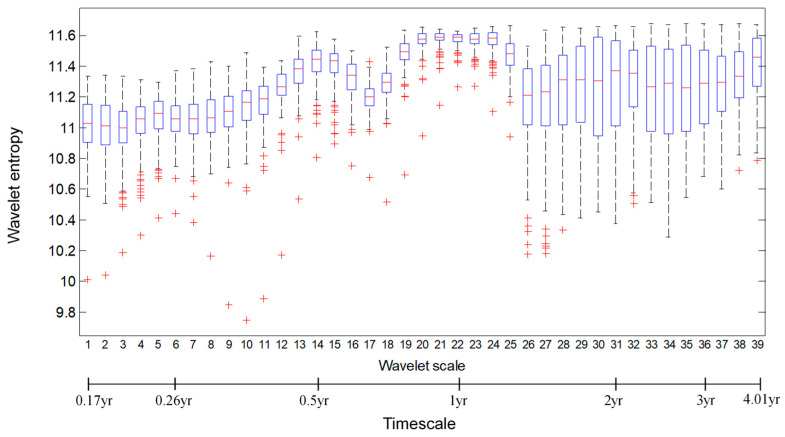
Boxplot for wavelet multi-scale entropy for a total of 169 grid cells. The bottom axes is the corresponding timescale for the 39 wavelet scales calculated by Equation (3), with the *s*_0_ 60-days (about 0.17 year), based on the daily step time series.

To display the wavelet-based entropy features for the soil moisture dynamics over the total 169 grid cells in the Xijiang River basin, Figure 4 summarizes the entropy variations for each of the 39 timescales (within 0.17–4.01 years) from 169 daily soil moisture time series. It is observed that the high entropy value with less variations is located around the 0.5-year and 1-year timescales, which indicates the soil moisture dynamics are still dominated by the annual and seasonal variability with less difference across the whole Xijiang basin area. Meanwhile, the high variations of entropy occurred at less than the 0.5-year and around the 2-year timescales, which demonstrates the local differences of the embedded short-term and long-term disorder characteristics over these timescales. These differences emphasize the importance of regional hydrological regionalization, i.e., the segments classification on the soil moisture dynamics for the Xijiang River basin in this study.

### 3.2. Hydrological Regionalization

**Figure 5:**
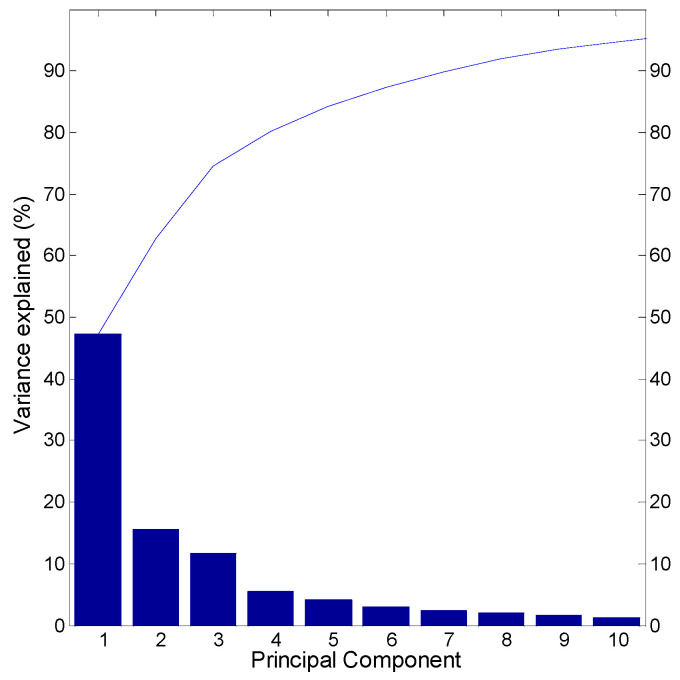
The scree plot of the first ten principal components of the wavelet-based multi-scale entropy.

**Figure 6:**
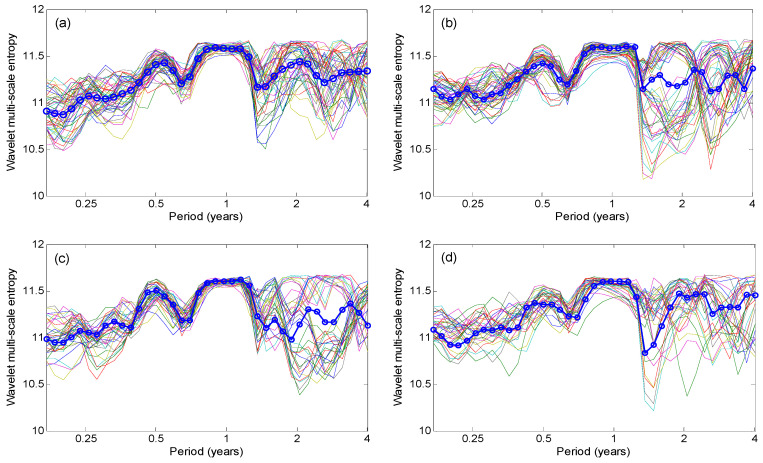
The obtained multi-scale wavelet entropy among the 169 grid cells for (**a**) the first cluster, (**b**)the second cluster, (**c**) the third cluster, and (**d**) the fourth cluster over the Xijiang River basin (The solid line with the circle is the average one for each cluster).

To reveal the similarity of multi-scale soil moisture dynamics in different grid cells in the Xijiang River basin in South China, the hydrological regionalization is performed based on wavelet-based multi-scale entropy of soil moisture data by employing PCA method. Figure 5 shows that the explained variability embedded in all the wavelet-base entropy for 39 timescales by the first 10 principle components, which accounts for about 94.67% of the total variability (see Table 1). The number of clusters are determined by both the Scree plot and the Kaiser’s rule. The first four principle components (which explains 80.14% of the total variability) are used to reflect the total entropy variability presented across 39 timescales over the studied basin. Figure 6 shows the clustered wavelet-based entropy variabilities of different grid cells for the obtained four coherent modes (the bold curve). The hydrological similarity in terms of multi-scale soil moisture dynamics is observed in the different clusters.

**Figure 7:**
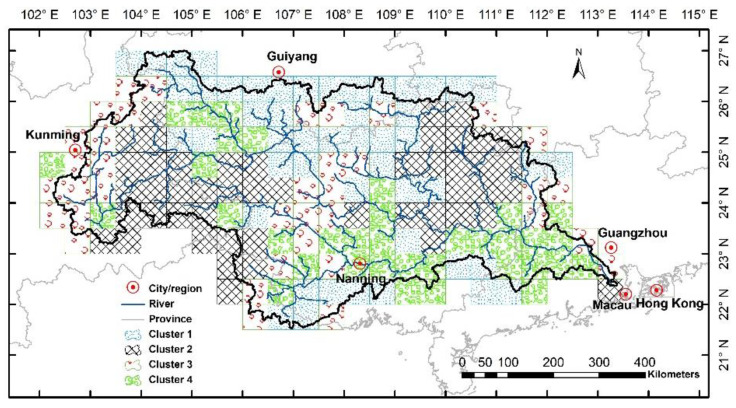
The geographical distribution of the classified grid cells based on the multi-scale wavelet entropy information.

The geographical distributions of the four coherent wavelet-based entropy modes are shown in Figure 7. For the first cluster, it mainly locates at the northern and southern edges of the Xijang River basin, where the headwater regions with high-slope mountainous areas are. The second cluster of grid cells shows the two contiguity regions, which are geographically separated, but both are middle and lower reaches of three sub-basins (i.e., Nanpan, Guihe, and Liujiang River basins). The third cluster is around the main river channels of the Xijiang River basin, especially for the lower reaches of the whole Xijiang River basin. The fourth cluster has a common feature with a relatively low-slope region, including both headwater regions (i.e., Napan and Guihe River basin) and the flat area (i.e., Hongshui River basin). Therefore, the hydrological regionalization of soil water dynamics on wavelet-based entropy reflects both geographic contiguity and similar soil water-related response characteristics.

## 4. Discussion

Hydrological regionalization is an important step towards the hydrological applications of the similar approaches or estimations of similar parameter values, especially when a large-scale basin area is involved. One distinguished feature of the present study is that the regionalization is based on wavelet multi-scale entropy information of simulated soil water data. Although the Xijiang River basin in South China has abundant precipitation, drought events frequently occurred due to uneven distributions in both the seasonal and spatial aspects. This irregularity is further aggregated by the diversified land surface characteristics. However, we can still identify the coherent regions, as the obtained results in this study.

The clusters of soil moisture dynamics, based on wavelet-based multi-scale entropy will help us develop similar approaches to short-term or long-term drought forecasting, as the soil water in the grid cells located in the same clustered region may have consistent variability and disorder features across different timescales. Furthermore, the response features of agricultural drought (mainly reflected by soil water) to metrological drought (mainly represented by precipitation) are also grouped based on the obtained results, which are certainly favorable for the formation of drought mitigation strategies in the basin. There are also some alternate watershed regionalization methods [37,38,39]. The joint entropy approach could be employed, when we want to retrieve vegetation growth patterns from multiple variables (e.g., soil moisture, precipitation, and temperature) [39]. Furthermore, the analyzed variables (such as streamflow, precipitation, and land-cover) may interact differently in time and space, and the entropy-based index and k-mean clustering may be employed for the spatiotemporal analysis [37,38]. In the present study, we emphasized the multi-scale variability and disorder features using the wavelet-based multi-scale entropy method.

## 5. Conclusions

In the Xijiang River in South China, water resources are increasingly important to fulfill the regional water demands, in the face of extreme hydrological events and increasing water requirement for the large cities around the lower reaches of the basin. For a large-scale basin, such as the case of the Xijiang River basin here, it is difficult to perform comprehensive hydrologic regionalization, due to the lack of land surface fluxes and the capture of the multi-scale variability features. This study proposed a framework (shown in Figure 2), mainly including long-term effective hydrological modeling, multi-scale wavelet entropy analysis, and cluster analysis. It demonstrates the hydrological regionalization in terms of the variability and disorder features of soil water dynamics, with a 0.5° × 0.5° grid cell basis over the basin, which will provide clues to regional water resource management. 

The continuous wavelet transform is performed on daily top-1 m soil moisture data over 39 timescales (within 0.17–4.01 years) for 169 grid cells. The multi-scale variability is then revealed by both the local wavelet power and global wavelet power. The entropy information of the wavelet-based power becomes a measure of randomness of the given grid cell-based soil moisture time series at different timescales. The dominant timescale bands for the entropy features of soil moisture variability are identified at seasonal and annual timescales, but the diversified disorder features of them are found around less than the 0.5-year and around the 2-year timescales, which indicates the necessity of the hydrologic regionalization of soil moisture dynamics in the region.

The hydrological regionalization of soil water dynamics are subsequently carried out, in terms of the multi-scale wavelet entropy, using the principal component analysis. The four distinct modes were obtained, which can account for 80.14% of the embedded variability in the wavelet power of the soil water time series over 169 grid cells in the basin. The clusters of different coherent modes are geographically close or apart, and different clusters show the distinct geographical characteristics and soil water response features. The hydrological regionalization of soil water dynamics is favorable to the selection of modeling/forecasting approaches and relevant model parameter estimation, especially for a large-scale basin. Meanwhile, it could be useful information during drought mitigation processes.

## Figures and Tables

**Table 1:** Eigenvalues, proportions of variance and cumulative proportions of variance for the first ten principal components.

Measures	PC1	PC2	PC3	PC4	PC5	PC6	PC7	PC8	PC9	PC10
Eigenvalues	79.81	26.26	19.91	9.46	7.01	5.21	4.23	3.41	2.73	1.97
Proportions of variance	47.22	15.54	11.78	5.60	4.15	3.08	2.50	2.02	1.62	1.17
Cumulative proportions of variance	47.22	62.76	74.54	80.14	84.29	87.37	89.87	91.88	93.50	94.67
